# Effects of LDL Receptor Modulation on Lymphatic Function

**DOI:** 10.1038/srep27862

**Published:** 2016-06-09

**Authors:** Andreea Milasan, François Dallaire, Gaétan Mayer, Catherine Martel

**Affiliations:** 1 Department of Medicine, Faculty of Medicine, Université de Montréal, Montreal, Quebec, Canada; 2 Montreal Heart Institute, Montreal, Quebec, Canada; 3 Laboratory of Molecular Cell Biology, Montreal Heart Institute Research Center, Quebec, Canada; 4 Faculty of Pharmacy, Université de Montréal, Montreal, Quebec, Canada

## Abstract

Atherosclerosis is driven by the accumulation of immune cells and cholesterol in the arterial wall. Although recent studies have shown that lymphatic vessels play an important role in macrophage reverse cholesterol transport, the specific underlying mechanisms of this physiological feature remain unknown. In the current report, we sought to better characterize the lymphatic dysfunction that is associated with atherosclerosis by studying the physiological and temporal origins of this impairment. First, we assessed that athero-protected Pcsk9^−/−^ mice exhibited improved collecting lymphatic vessel function throughout age when compared to WT mice for up to six months, while displaying enhanced expression of LDLR on lymphatic endothelial cells. Lymphatic dysfunction was present before the atherosclerotic lesion formation in a mouse model that is predisposed to develop atherosclerosis (Ldlr^−/−^; hApoB100^+/+^). This dysfunction was presumably associated with a defect in the collecting lymphatic vessels in a non-specific cholesterol- but LDLR-dependent manner. Treatment with a selective VEGFR-3 agonist rescued this impairment observed early in the onset of this arterial disease. We suggest that LDLR modulation is associated with early atherosclerosis-related lymphatic dysfunction, and bring forth a pleiotropic role for PCSK9 in lymphatic function. Our study unveils new potential therapeutic targets for the prevention and treatment of atherosclerosis.

Atherosclerosis is driven by the accumulation of cholesterol in the artery wall, which triggers a maladaptive immune response in which macrophages play a prominent role. Circulating low-density lipoprotein-cholesterol (LDL-C) accumulates in the artery wall of blood vessels and leads to premature atherosclerosis^1^. LDL receptors (LDLR) are present on the outer surface of many types of cells^2^. Whereas macrophage LDLR has a major role in the uptake of native, unmodified LDL, it is involved in lesion development and foam cell formation *via* lipoprotein metabolism regulation^3^. LDLR expression on hepatocytes is crucial for the clearance of circulating LDL and its precursors, intermediate-density lipoproteins (IDL) and very-low density lipoproteins (VLDL)^4^. Apolipoprotein B100 (ApoB100) is the apolipoprotein and ligand of LDLR found in lipoproteins synthesized by the liver, and is the sole protein of LDL^4^. Proprotein convertase subtilisin/kexin type 9 (PCSK9) is a well-established down-regulator of LDLR, which acts by binding the receptor and causes its lysosomal degradation in cells^5^,^6^. PCSK9 prevents ApoB100 degradation, either directly^7^ or through a neutralizing effect on LDLR activity^8^. Since PCSK9 interferes with the clearance of LDL-C from the blood, its loss-of-function mutations are associated with up to 85% lower plasma LDL-C levels^9^ and offer significant protection from coronary artery disease (CAD), like atherosclerosis^10^. Contrarily, familial hypercholesterolemia is a genetic disorder characterized by severe elevation of the plasma levels of LDL, a result indicative of reduced function of LDLR or ApoB, or gain-of-function mutations in PCSK9^11^,^11^

Contrarily, high levels of high-density lipoprotein (HDL) is believed to reduce cardiovascular risk, mainly through its role in macrophage reverse cholesterol transport (mRCT), promoting cholesterol removal from plaque and its eventual excretion by the liver and/or the intestines^13^,^14^,^15^. In order to halt atherosclerosis progression and decrease the prevalence of CAD, emphasis has logically been put on improving this physiological process. However, most of the different treatments tested to increase HDL levels did not demonstrate any clinical benefits, and did not lead to improved mRCT or decreased CAD^16^,^17^,^18^. Consequently, such conclusions made the scientific community re-think the way they approach the development of therapies aimed to increase mRCT and favourably modulate atherosclerosis.

PCSK9 inhibition has an excellent safety profile in clinical trials and promises to provide a well-tolerated and effective therapeutic measure against coronary heart disease^19^. It could act *via* its direct modulation of LDL-C uptake by the liver, but could also potentially be associated with pleiotropic effects. Whereas most effects of PCSK9 are described based on its hepatic origin, one of the roles of PCSK9 that is under growing investigation is its presence and effect in atherosclerotic lesions. Giunzioni *et al.* showed for the first time that this PCSK9 expression directly influences atherosclerotic plaque composition with no changes in serum cholesterol levels^20^, concurring with other studies suggesting that extra-hepatic tissues could significantly contribute to PCSK9 production and could potentially regulate LDLR expression^21^,^22^. PCSK9 found in atherosclerotic lesions has been suggested by Ferri *et al*. to be mostly produced by vascular cells, as cultured smooth muscle cells have been reported as the only vascular cells expressing and secreting functionally active PCSK9^23^.

An additional new therapeutic target in atherosclerosis has recently arisen. In a recent study, the lymphatic system has been identified as a novel prerequisite player in the removal of cholesterol out of the atherosclerotic lesion by mRCT^24^. It has been reported that without a functional lymphatic network, cholesterol cannot leave the artery wall and might potentially aggravate the disease. Accordingly, it is now suggested that cholesterol leaves tissues and reaches the bloodstream by first entering lymphatic vessels (LVs), putting forward a new integrated model for mRCT^24^,^25^,^26^. The blood vasculature and the lymphatic system are parallel and interdependent networks^27^. In contrast to the blood vasculature, the lymphatic vascular network is an open, unidirectional and low-pressure vascular system. Lymphatic development and regulation are dependent mostly upon VEGF-C/D and the receptor VEGFR-3^28^. The LVs are composed of two different entities, bearing distinct but complementary roles. Lymph is first absorbed through thin-walled and blind-ended initial lymphatics (also called lymphatic capillaries), which are highly permeable and are constituted of specialized, discontinuous “button-like” junctions between endothelial cells^29^. Expression of lymphatic vessel hyaluronan receptor 1 (LYVE-1) on lymphatic endothelial cells (LEC) and absence of smooth muscle cells (SMC), are characteristics of the initial lymphatics^29^. Next, lymph moves from the initial lymphatics into collecting LVs, the entities responsible of maintaining lymph flow through contraction of units called lymphangions. Collecting LVs have bi-leaflet valves between the contractile units in order to prevent back flow. They are also characterized by a basement membrane, podoplanin expression, down-regulation of LYVE-1 expression, continuous “zipper-like” cell-cell junctions and a discontinuous SMC layer^30^. Lymph is propelled away from the periphery most of the time against a hydrostatic pressure gradient primarily *via* the phasic and synchronized contractions of the lymphangions, mediated by the intrinsic contractility of SMC, the contraction of surrounding skeletal muscles, and arterial pulsations^31^,^32^,^33^,^34^,^35^.

In the past decade, tremendous progress has been made to better characterize the interplay between the lymphatic network and chronic inflammatory diseases^36^, including atherosclerosis^37^. Studies analyzing the morphology of LVs in the artery wall allowed for insights into their associations with atherosclerosis. In animal models, LVs have been observed in the adventitia of the artery wall^38^. In fact, Xu *et al*. demonstrated the presence of LVs within the adventitia of the artery wall to be an important factor for the draining of local inflammatory cells and cytokines from peripheral tissues^39^. In a clinical setting, Drozdz *et al*. took interest in the presence of lymphatics in the adventitia of the internal carotid artery in humans and showed that the number of adventitial LVs increases with the severity of atherosclerosis measured as intimal thickness^40^. Along the same path, Vuorio *et al*. published that lymphatic impairment worsened the atherosclerosis plaque formation in atherogenic LDLR^−/−^/ApoB^100/100^ mice crossed with transgenic mice having lymphatic localized insufficiency, and analyzed the effects of the absence of lymphatics on lipoprotein metabolism and atherosclerosis^41^. This group observed a positive correlation between increased atheroma formation and the absence of LVs. It is now well established that LVs are more abundant in the adventitial layer of the arterial walls in both animals and humans^42^. Taken together, while it is now well accepted that lymphatic dysfunction is associated with atherosclerosis, the specific underlying mechanisms explaining this improper transport remain unknown.

In the present study, we sought to better characterize the lymphatic dysfunction that is associated with the onset and progression of atherosclerosis. Lymphatic function is now linked to atherosclerosis, and LDLR modulation plays a central role in CAD. Therefore, in this report, using athero-protected Pcsk9^−/−^ mice^43^, we studied PCSK9 potential pleiotropic effects on lymphatic function. We herein demonstrate that absence of PCSK9 and thus increased LDLR protein expression has a beneficial effect on collecting LV function throughout age when compared to wild type (WT) mice for up to six months, while displaying high expression of LDLR on LEC. Then, using Ldlr^−/−^; hApoB100^+/+^, we assessed the physiological and temporal origins of the defective lymph transport observed in atherosclerosis. We showed that lymphatic dysfunction was present before the atherosclerotic lesion formation, and that this dysfunction was presumably associated with a defect in the collecting LVs in a non-specific cholesterol- but LDLR-dependent manner. Furthermore, systemic treatment with a selective VEGFR-3 agonist rescued this lymphatic transport impairment observed early in the onset of this arterial disease. Altogether, our results bring forward a new pleiotropic role for PCSK9 in lymphatic function and unveil new potential therapeutic targets for the prevention and treatment of atherosclerosis.

## Results

### Lymphatic vessel function is enhanced in Pcsk9^−/−^ mice

**Figure 1:**
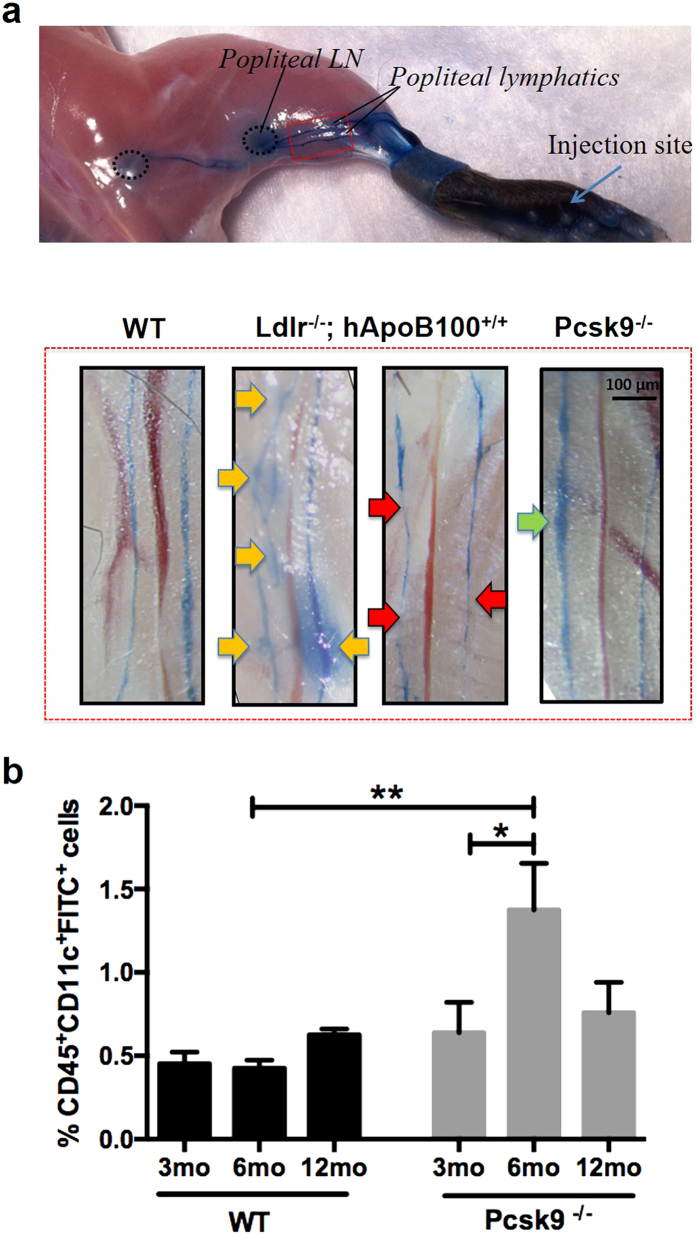
Lymphatic function is enhanced in Pcsk9^−/−^ mice. **(a)** Lymphatic vascular function was assessed following Evans Blue dye intradermal injection in the footpad of 6-month-old WT, Pcsk9^−/−^ and Ldlr^−/−^; hApoB100^+/+^ mice. After 30 minutes, lymphatic vessels were visualized using Stereo Discovery V8. Pictures were taken by Canon Rebel XSI. Pcsk9^−/−^ mice show a higher intensity of Evans Blue dye within the vessel (green arrow), and clear surroundings. In contrast, Ldlr^−/−^; hApoB100^+/+^ mice demonstrate interrupted Evans Blue dye presence (red arrows), as well as extravasated dye from these vessels (yellow arrows). Similar results were obtained in at least three repeated experiments. **(b)** Dendritic cell migration, examined 18h after a contact sensitization assay (FITC painting) was measured by quantification of %CD45^+^CD11c^+^FITC^+^cells from lymph nodes of 3-, 6- and 12- month-old WT and Pcsk9^−/−^ mice. Experiments were performed using 5–14 mice per experimental group (mean ± SEM). Analysis was performed using a BD LSRII flow cytometer. *p < 0.05 and **p < 0.01.

As PCSK9 targets and mechanisms of action are now known not to be solely confined to the liver, we sought to investigate its effect on lymphatic function. We herein hypothesized that knocking out PCSK9, and thus decreasing circulating LDL levels throughout age when compared to WT, may positively modulate lymphatic transport. To address this, we first assessed the capacity of popliteal LVs to carry Evans blue (EB) dye from the initial lymphatics located in the dermis of the foot pad up to the collecting lymphatics and the corresponding draining popliteal lymph node (LN) (Fig. 1a, upper panel; Supplementary Fig. S1). Intradermal injection of EB dye revealed that the dye intensity was greater within the dominant^44^ collecting vessel of Pcsk9^−/−^ mice, with no or little interruption on its path (Fig. 1a, lower panel, green arrow *vs*. red arrows). This observation suggests that collecting lymphatic vessel function was improved in Pcsk9^−/−^ mice when compared to atherosclerosis-prone Ldlr^−/−^; hApoB100^+/+^ and even WT mice. In addition, less extravasated EB dye was detected in the surrounding adipose tissue of Pcsk9^−/−^ mice when compared to that of Ldlr^−/−^; hApoB100^+/+^ mice, signifying a more proper collecting LV function.

As a complementary measure of lymphatic function, we evaluated the efficiency of LVs to transport dendritic cells (DC) from the peripheral tissue to the corresponding draining LNs, using the well-described FITC painting assay^45^. Similar to the improved EB dye transport, the migration of skin dendritic cells (CD45^+^CD11c^+^FITC^+^) in Pcsk9^−/−^ mice (Fig. 1b) was improved when compared to WT mice at 6 months. Analysis of the back skin from that same region (Supplementary Fig. S2a) revealed that the adipose tissue layer tends to be thinner in Pcsk9^−/−^ mice when compared to WT mice, with a marked difference in 3-month old mice (Supplementary Fig. S2b). As lymph is rich in lipoproteins, we have measured HDL levels in lymph of Pcsk9^−/−^ animals and found that, similarly to plasma (Supplementary Fig. S3a)^46^, their levels were lower compared to that of WT mice at 6 months of age (Supplementary Fig. S3b).

### Initial lymphatic vessel morphology and number are unchanged in Pcsk9^−/−^ mice

**Figure 2:**
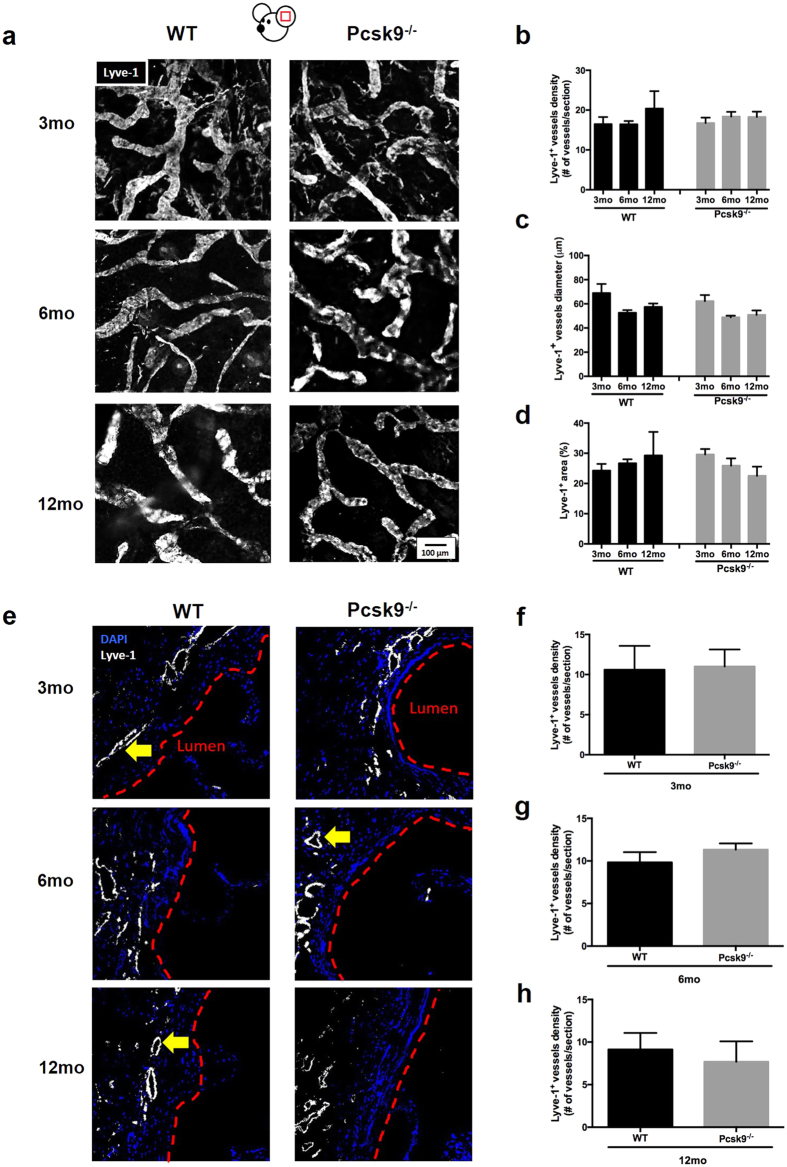
Initial lymphatic vessel number, diameter, and area are unchanged in Pcsk9^−/−^ mice. **(a)** Immunofluorescence of initial lymphatic vessels (Lyve-1^+^) in the ear dermis of 3-, 6- and 12-month-old WT and Pcsk9^−/−^ animals. Quantification of the **(b)** number **(c)** diameter and **(d)** surface area of Lyve-1^+^ vessels in the ear dermis. Experiments were performed using 5–9 mice per experimental group. **(e)** Immunofluorescence of initial lymphatic vessels (Lyve-1^+^) and DAPI in the aortic sinus adventitia in 3-, 6- and 12-month old WT and Pcsk9^−/−^ mice. Only Lyve-1^+^ vessels of any continuous tube-like shapes formed of Lyve-1^+^ CD68^−^ were included, as indicated by the yellow arrows. Quantification of the number of Lyve-1^+^ vessels/aortic section at **(f)** 3-, **(g)** 6- and **(h)** 12-month mice. Experiments were performed with 3–6 mice per experimental group (mean of 2–3 measurements/slide ± SEM). Pictures were taken using Fluoview FV10i confocal microscope (Olympus). All image handling was performed using ImageJ software.

As hypercholesterolemia-associated lymphatic dysfunction in 16-week-old apoE^−/−^ mice is associated with initial lymphatic hyperplasia^47^, we conversely sought to investigate whether the absence of PCSK9 would be associated with morphological changes within initial lymphatics. We first assessed initial lymphatic vessel (Lyve-1^+^) morphology and density (Fig. 2a) by looking at their number (Fig. 2b), diameter (Fig. 2c) and total surface area occupied by the vessels (Fig. 2d) in the mouse ear dermis. No significant changes were observed in the athero-protected Pcsk9^−/−^ mouse model when compared to WT mice in an age-dependent manner. In order to confirm our finding in the artery wall, we investigated the presence of initial lymphatics in the adventitia of the aortic sinus (Fig. 2e), a blood vessel layer where LVs have been consistently observed^26^. Once again, no difference was seen at any age when looking at the morphology or density of Lyve-1^+^ vessels (Fig. 2f–h).

### LDLR is expressed on lymphatic endothelial cells of collecting lymphatic vessel

**Figure 3:**
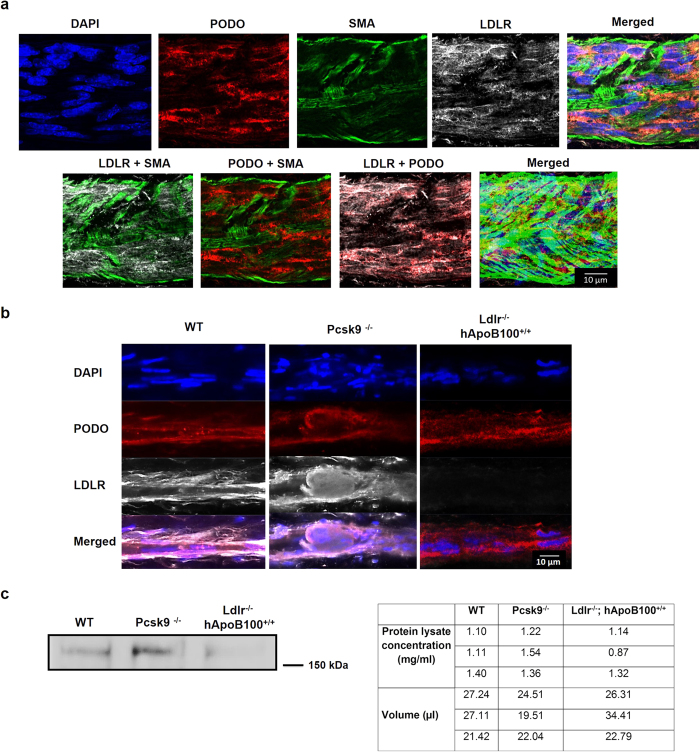
LDLR is expressed on lymphatic endothelial cells of collecting lymphatic vessel and its level is modulated by PCSK9. **(a)** LDLR expression on smooth muscle cells (smooth muscle actin, SMA) and LEC was detected by immunofluorescence of DAPI, podoplanin (PODO) and LDLR in longitudinally imaged single plan in popliteal lymphatic collecting vessels of WT mice. In the lower right panel, z-stacks were acquired and deconvolved. **(b)** Immunofluorescence of longitudinally imaged lymphatic collecting vessel single plan of WT, Pcsk9^−/−^ and Ldlr^−/−^; hApoB100^+/+^ animals. Images were acquired with an LSM 710 Confocal Microscope (Zeiss) equipped with a 63×/1.4 oil dic objective. **(c)** Popliteal lymphatic collecting vessels were isolated and digested. LDLR protein expression was assessed by Western Blot in WT, Pcsk9^−/−^ and Ldlr^−/−^; hApoB^+/+^ mice. LDLR protein was observed at 160 kDa. The table on the right confirms equal protein loading.

Based on our findings that lymphatic function is improved in Pcsk9^−/−^ mice without apparent modulation in initial lymphatic morphology or growth, we sought to investigate whether collecting LVs could thus be responsible for the related lymphatic gain-of-function observed in this Pcsk9^−/−^ mouse model. To begin with, we hypothesized that LDLR *per se* could act as an important modulator in collecting lymphatic vessel function, as it would be drastically increased on LVs compared to WT mice. Our initial step was to detect the presence of LDLR on collecting LVs, to then attest its modulation through PCSK9. Therefore, for the first time to our knowledge, we have shown that LDLR was expressed on popliteal collecting LVs (Fig. 3a). Immunofluorescence was performed and attested the presence of LDLR on podoplanin^+^ LECs, but to a lesser extent on smooth muscle cells (SMC, Fig. 3a). Immunofluorescence (Fig. 3b) and Western blots performed with equal amount of protein loading (Fig. 3c) revealed that LVs of Pcsk9^−/−^ mice displayed increased LDLR protein expression level compared to WT and Ldlr^−/−^; hApoB100^+/+^ mice.

### PCSK9 is present in circulating lymph

**Figure 4:**
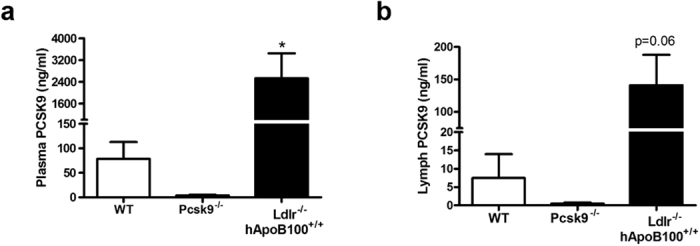
Circulating PCSK9 levels in plasma and lymph. Levels of circulating PCSK9 were measured in **(a)** plasma and **(b)** lymph of WT, Pcsk9^−/−^ and Ldlr^−/−^; hApoB100^+/+^ mice. Experiments were performed using 5−9 mice per experimental group. P-values compare WT vs. Ldlr^−/−^; hApoB100^+/+^ animals. p < 0.05.

Our data are bridging lymphatic function to LDLR modulation on collecting LECs through PCSK9. Therefore, we next conversely investigated whether decreased LDLR levels *per se* could be a premise to collecting LV dysfunction. As our results showed that LDLR is present on LEC (Fig. 3a) and that this protein expression is increased in the absence of PCSK9 (Fig. 3b,c), we sought to determine the levels of circulating PCSK9 in lymph. Proportionally to plasma PCSK9 levels, lymph isolated from the thoracic duct of Ldlr^−/−^; hApoB100^+/+^ mice tended to contain more circulating PCSK9 than WT animals (Fig. 4a,b; lymph isolated from Pcsk9^−/−^ mice served as control). These results suggest that the modulation of LEC LDLR levels could be, at least in part, attributable to the presence of circulating PCSK9 in lymph.

### Lymphatic dysfunction appeared before atherosclerosis onset and worsened during its progression in Ldlr^−/−^; hApoB100^+/+^ mice

**Figure 5:**
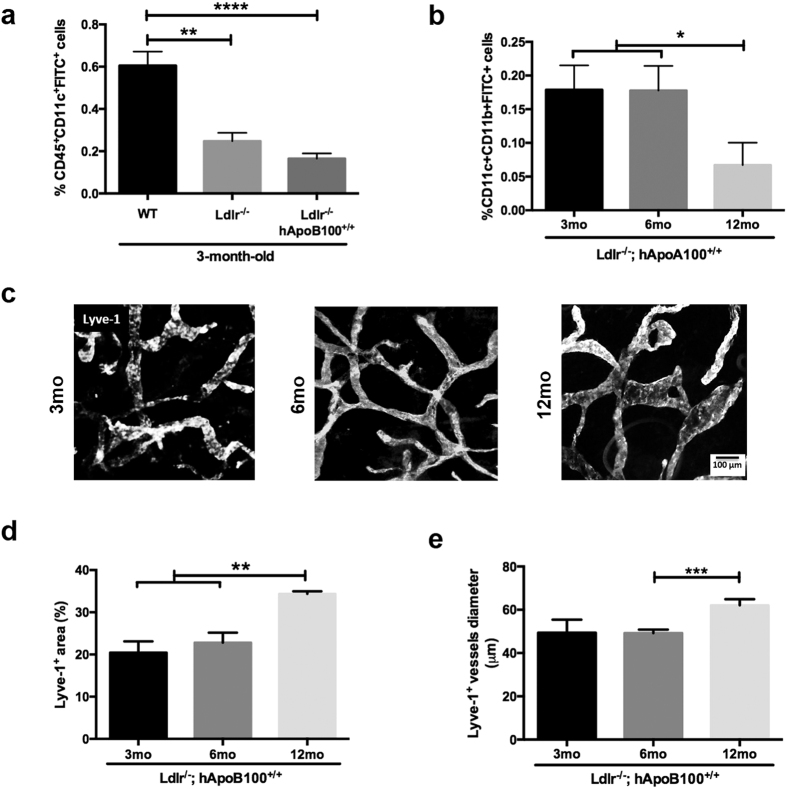
Lymphatic function in the onset and progression of atherosclerosis in Ldlr^−/−^; hApoB100^+^/^+^ mice. Dendritic cell migration was assessed 18 h after FITC painting by quantifying percent of CD45^+^ CD11c^+^ FITC^+^ cells from digested lymph nodes of **(a)** 3-month-old WT, Ldlr^−/−^ and Ldlr^−/−^; hApoB100^+^/^+^ mice, and **(b)** 3-, 6- and 12-month-old Ldlr^−/−^; hApoB100^+/+^ mice. Experiments were performed with 5–14 mice per experimental group. **(c)** LYVE-1 immunostaining was examined in ear whole mounts from Ldlr^−/−^; hApoB^+/+^ mice at ages of 3-, 6- and 12-month-old. Quantification of the **(d)** total surface area of Lyve-1^+^ vessels and **(e)** the diameter of initial lymphatics in the ear dermis. Experiments were performed with 4–9 mice per experimental group (mean of 5 measurements/slide ± SEM). Pictures were taken using Fluoview FV10i. All image handling was performed using ImageJ software. *p < 0.05, **p < 0.01 and ***p < 0.001.

Atherosclerosis-prone Ldlr^−/−^; hApoB100^+/+^ mice are severely dyslipidemic, exhibit premature endothelial cell dysfunction, oxidative stress and inflammation^48^,^49^,^50^.They spontaneously develop aortic atherosclerotic lesions after 4 months while on regular chow diet. Before that age, they do not have atherosclerotic lesions, but as they get older, they become increasingly atherosclerotic (Supplementary Fig. S4a^49^. We have decided to use this specific mouse model as it resembles to the human atherosclerotic phenotype with a similar lipoprotein profile, due in part by the addition of the human ApoB100 transgene. These mice represent a suitable model to study in further details factors that could lead to atherosclerosis or impact its progression. Lymphatic transport assays revealed a significant lymphatic function impairment in 3-month-old Ldlr^−/−^; hApoB100^+/+^ mice (Fig. 5a) that are not yet bearing atherosclerotic lesions (Supplementary Fig. S4a,c,d or accumulation of macrophages in the artery wall (Supplementary Fig. S4b,e. To support this, we showed that, in contrast to Pcsk9^−/−^ mice (Fig. 1a), Ldlr^−/−^; hApoB100^+/+^ mice displayed an EB dye flow that was either interrupted (red arrows) or extravasated (yellow arrows) from the collecting LVs.

As these mice are severely dyslipidemic even at a young age (Supplementary Fig. S5, we wanted to test whether this high plasma cholesterol content *per se* would be reflected by an enhanced lymphatic transport defect. To test this, we have measured lymphatic function in both young (3-month-old) pre-atherosclerotic Ldlr^−/−^; hApoB100^+/+^ mice and Ldlr^−/−^ mice. As total circulating cholesterol content of Ldlr^−/−^ mice is known to be lower than half the concentration present in the Ldlr^−/−^; hApoB100^+/+^ mice (~200 mg/ml vs. ~700 mg/ml)^49^,^51^,^52^, one could expect to see an even more impaired lymphatic function in the latter group. However, as a premise of the effect of LDLR *per se* on lymphatic function as a pro-atherosclerotic factor, we observed no significant difference between the two groups (Fig. 5a). Whereas this lymphatic dysfunction was stable for up to 6 months, it drastically worsened in the twelfth month of age (Fig. 5b), concurring with age-related lymphatic dysfunction^53^. As Pcsk9^−/−^ mice abundant in LDLR display improved lymphatic transport and as 3 month-old pre-atherosclerotic Ldlr^−/−^; hApoB100^+/+^ mice lacking LDLR showed a lymphatic dysfunction that was exacerbated in parallel to lesion formation, these results reinforce the idea that the LDLR *per se* could play a direct role on lymphatic function.

We next sought to investigate by immunofluorescence (Fig. 5c) whether this pre-atherosclerosis related-lymphatic dysfunction would also be associated with morphological changes within initial lymphatics. Twelve-month old Ldlr^−/−^; hApoB100^+/+^ mice displayed increased total Lyve-1^+^ area (Fig. 5d) and hyperplasic initial LVs (Fig. 5e) compared to 3- and 6-month old mice, reflecting in part the chronologically increased DC transport impairment. However, as no initial lymphatic hyperplasia is observed at 3 months despite our previously reported defects in lymphatic transport *per se*, these results suggest that initial LV function is seemingly not responsible for early atherosclerosis-related lymphatic dysfunction.

### Systemic treatment with selective VEGFR-3 agonist rescues lymphatic function in pre-atherosclerotic Ldlr^−/−^; hApoB100^+/+^ mice

**Figure 6:**
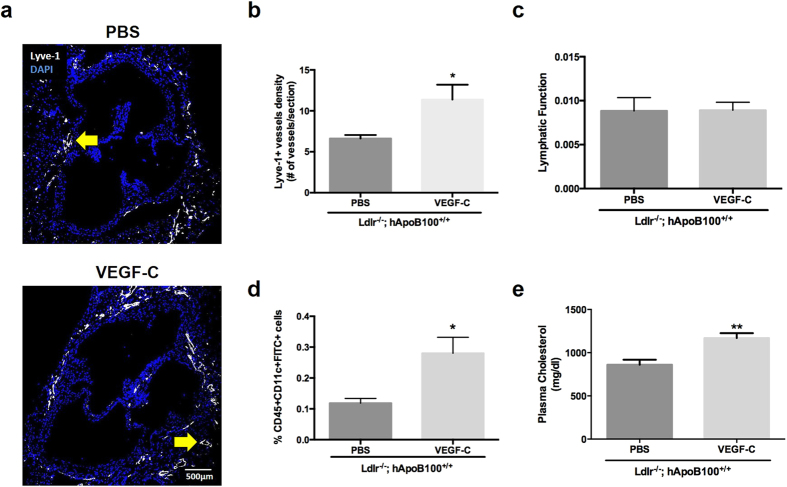
VEGF-C 152s treatment rescues lymphatic cellular transport in pre-atherosclerotic Ldlr^−/−^; hApoB100^+^/^+^ mice. **(a)** Immunofluorescence of initial lymphatic vessels (Lyve-1^+^) and DAPI of 3 month-old PBS-treated (control) and VEGF-C 152s-treated Ldlr^−/−^; hApoB100^+/+^ mice. Only Lyve-1^+^ vessels of any circular shape were included, as indicated by the yellow arrows. Quantification of the **(b)** number of Lyve-1^+^ vessels/aortic section. Experiments were performed using 5–7 mice per experimental group. Pictures were taken using Fluoview FV10i. All image handling was performed using ImageJ software. **(c)** Lymphatic molecular transport was assessed by Cy5-labelled Dextran (70 kDa) injection in the ear dermis of PBS- and VEGF-C 152s-treated Ldlr^−/−^; hApoB100^+/+^ mice mice. **(d)** Percent of CD45^+^CD11c^+^ FITC^+^ cells in skin-draining lymph nodes of 3 month-old PBS-treated (control) and VEGF-C 152s-treated Ldlr^−/−^; hApoB100^+/+^ mice. **(e)** Total cholesterol was measured in plasma of Ldlr^−/−^; hApoB100^+/+^ mice treated with VEGF-C 152s or PBS-control. Experiments were performed using 4–5 mice per experimental group (mean ± SEM) *p < 0.05.

VEGF-C was proven to reverse hypercholesterolemia-associated lymphatic dysfunction in apoE^−/−^ mice and to stimulate lymphatic pumping *ex vivo* in a model of rat mesenteric lymphatics by a VEGFR-3-dependent mechanism^31^. Therefore, as our results pointed out that LDLR mediated-lymphatic function modulation would most likely be due to an effect on collecting LVs, we then investigated whether and how lymphatic dysfunction can be restored in young Ldlr^−/−^; hApoB100^+/+^ mice. VEGF-C 152s is a point mutant that only binds to and activates signaling through VEGFR-3, and unlike wild type VEGF-C, is unable to bind VEGFR-2. VEGF-C treatment has previously been shown to restore lymphatic function in mice with established atherosclerosis^36^. We herein aimed to assess whether treatment with the specific VEGFR-3 agonist VEGF-C 152s could rescue lymphatic function before the onset of atherosclerosis in 3-month-old pre-atherosclerotic Ldlr^−/−^; hApoB100^+/+^ mice. Following treatment, the number of Lyve-1^+^ vessels in the adventitia of the aortic sinus (Fig. 6a) was significantly increased when compared to control (PBS-treated Ldlr^−/−^; hApoB100^+/+^) mice (Fig. 6b), despite no differences observed regarding dextran-Cy5 absorption by the initial lymphatics (Fig. 6c). However, a significant increase in lymphatic cellular transport was reported (Fig. 6d). Lastly, in order to exclude the fact that VEGF-C could have decreased total plasma cholesterol (TPC) and subsequently improve lymphatic function, TPC was measured in VEGF-C 152s-treated Ldlr^−/−^; hApoB100^+/+^ mice and control mice. To the contrary, our results showed that TPC was even significantly higher in VEGF-C 152s-treated mice compared to PBS-treated mice (Fig. 6e), suggesting that the beneficial effect of VEGF-C 152s on lymphatic function in the early stage of atherosclerosis is not related to decreased hypercholesterolemia.

## Discussion

Based on original data from the Framingham Study indicating that low plasma levels of HDL-C are associated with premature CAD^54^, increasing HDL levels has become an attractive approach aimed at better controlling related pathologies such as atherosclerosis. However, recent findings have met with disappointing clinical outcomes, emphasizing that atherosclerosis is a multifactorial disease and that many different avenues need to be considered in order to better control the atherosclerotic process and prevent CAD. In that context, a prerequisite role for the LVs in mRCT has been brought forward, suggesting that without a functional lymphatic network, atherosclerosis is exacerbated. In the present work, we shed light on the specific underlying mechanisms that could be the premise of the improper lymphatic transport observed in this arterial disease.

PCSK9 has emerging therapeutic roles in dyslipidemia-associated diseases, particularly atherosclerosis, and new evidence shows that LVs play a key role in controlling cholesterol efflux from peripheral tissues, such as the atherosclerotic lesion^24^. With this perspective, we here combine recent knowledge governing the identification of two attractive new therapeutic targets in atherosclerosis, namely PCSK9 and the lymphatic system, and sought to characterize their possible interaction with the ultimate goal of limiting the disastrous consequences related to this pathology. In this study, we aimed to better delineate the effects of the modulation of LDLR, particularly through PCSK9 inflection, on lymphatic function.

On top of its positive effect on plasma cholesterol uptake by LDLR, our results suggest that lymphatic function also greatly benefits from downregulation of PCSK9. The limited subcutaneous adipose tissue accumulation we observed in Pcsk9^−/−^ mice opposes the increased visceral adipogenesis previously reported that is most likely due to VLDLR^55^ and/or CD36^56^, and their role in triglycerides regulation. Whereas the regulation of the latter could be considered in our findings, we cannot exclude a potential role of the LVs in the clearance process of the adipose tissue from peripheral sites, such as the skin. Using two independent techniques, our first set of results demonstrates that lymphatic function is enhanced in Pcsk9^−/−^ mice, when compared to WT mice. This effect peaks on the 6^th^ month of age, and seems to diminish by the 12^th^ month, suggesting that PCSK9 inhibition could abrogate the aging-related lymphatic dysfunction up to a certain stage. It was recently documented by Dixon’s group that a preferential drainage pattern in LVs is possible through what they call “dominant vessels”, and in a smaller proportion in “non-dominant vessel”^44^. They bring forward the interesting hypothesis that dominant LVs are driven by extrinsic factors, while non-dominant vessels are under the influence of intrinsic contraction. The results obtained through the EB dye assay included in the present report further supports this affirmation.

We next investigated the morphology and number of initial lymphatics present in the ear dermis and in the adventitia of the aortic sinus of Pcsk9^−/−^ mice. Normal initial lymphatics, without visible changes in the morphology and number, at any age, were observed. Therefore, as we noted improvement in DC and EB dye transport, we believe that enhancement in LDLR levels may mostly affect collecting LVs rather than initial lymphatic function. Subsequently, it was necessary to demonstrate the ubiquitous nature of LDLR by assessing its presence on lymphatic endothelial cells of collecting LVs. Despite a reduced lymph flow, Le May *et al*. observed that PCSK9 expression impacts postprandial triglyceridemia^57^, another important cardiovascular risk factor. As they reported an increased presence of LDLR in the intestines, it should be taken into consideration that there might be an increase of LDLR at the surface of the lacteals (i.e. lymphatic vessels of the intestine) thus playing a role in lymphatic transport *per se*. To consolidate our findings, we have measured HDL levels in lymph of Pcsk9^−/−^ animals and found that their levels are much lower compared to that of WT mice at 6 months of age. This result could be an indication of an improved RCT capacity, as HDL is seemingly being transported more efficiently from the peripheral tissues toward the blood circulation^24^,^26^ in these athero-protected mice. Increased hepatic LDLR levels in Pcsk9^−/−^ mice are believed to accelerate clearance of circulating plasma ApoE-containing lipoprotein particles such as VLDL and HDL^46^, and hence we surmise that HDL particles are cleared faster from the lymph of Pcsk9^−/−^ mice.

Using atherosclerosis-prone Ldlr^−/−^; hApoB100^+/+^ mice that are severely dyslipidemic and predisposed to develop atherosclerosis by the age of 4 to 6 months^58^ even when fed regular chow diet^48^,^49^,^50^, we observed lymphatic dysfunction as early as 3-month-old mice, before any sign of lesion formation. The sinuous and leaking paths reflecting the flow of EB dye along the popliteal lymphatic vessel echoes a tangible dysfunction of the collecting LV. This observed lymphatic transport defect continues to worsen in concert with age, a phenomenon that was happening in conjunction with increased total Lyve-1^+^ area and hyperplasic initial LVs. Whereas aging^53^ and hypercholesterolemia^47^ can contribute to this enhanced lymphatic dysfunction, none of these factors can solely explain the early atherosclerosis-related lymphatic transport impairment. We herein demonstrated that this lymphatic dysfunction observed in the 3-month-old pre-atherosclerotic mice was apparently due to a lack of LDLR *per se*. Three-month-old Ldlr^−/−^ mice have a TPC that ranges between 200–370 mg/dl^49^,^51^,^52^, whereas TPC of the Ldlr^−/−^; hApoB100^+/+^ mice included herein is much higher (860 mg/dl). Despite this major difference in cholesterol levels, both 3-month-old Ldlr^−/−^ and Ldlr^−/−^; hApoB100^+/+^ mice depict similar impairment in lymphatic transport. These results further support the concept that lymphatic dysfunction observed in the early stage of atherosclerosis might be attributable to an LDLR-dependent collecting LVs malfunction. Ldlr^−/−^; hApoB100^+/+^ mice carry premature blood endothelial cell dysfunction^48^,^49^,^50^, a defect that we believe could be extrapolated to LEC. We hypothesize that the exacerbated lymph PCSK9 level observed in Ldlr^−/−^; hApoB100^+/+^ mice causes excessive LDLR degradation on LEC. This phenomenon possibly interferes with multiple cellular signaling pathways that could ultimately be involved in proper collecting LV function. A consequence could be the enhancement of endothelial nitric oxide synthase (eNOS) production, thus modulating oxidative stress. Therefore, overproduction of nitric oxide (NO) by the endothelium could act as a vasoactive agent to subsequently reduce lymphatic contractile activity^26^,^59^.

VEGF-C has been shown to reduce lymphedema by stimulating lymphangiogenesis^33^,^34^,^60^, and its binding to VEGFR-3 has been recently reported to alter the intrinsic and phasic pumping of collecting LVs^31^. Herein, we observed that 3-month-old pre-atherosclerotic Ldlr^−/−^; hApoB100^+/+^ mice treated with VEGF-C 152s displayed significantly less DC migration impairment compared to control mice. The capacity of the molecular tracer to be uptake by the initial lymphatics was unchanged between the two groups, confirming our hypothesis that the early atherosclerosis-related lymphatic dysfunction is most likely due to a defect in the collecting vessels rather than in the peripheral lymph uptake *per se*. As previously mentioned, hypercholesterolemia is a factor known to worsen lymphedema and lymphatic malfunction^47^. When we tested whether the positive effect of VEGF-C 152s could be attributable a decrease in circulating total cholesterol, we reported that VEGF-C 152s had the conversed effect of increasing TPC instead of decreasing it, therefore excluding this possibility. *In vitro* studies have revealed that VEGF-C/VEGFR-3 binding on LEC induces activation of PI3K/Akt and results in phosphorylation of eNOS^30^, thus affecting lymphatic contraction capacity. Like VEGF, VEGF-C has vasoactive- and endothelial barrier-altering properties^61^,^62^,^63^, and it has similarly recently been shown that its binding to VEGFR-3 can alter the intrinsic and phasic pumping of collecting LVs^31^. Whereas our data suggest that early atherosclerosis-related lymphatic dysfunction could logically be abrogated through positive upregulation of LEC LDLR protein expression, we also show that VEGF-C treatment can restore this impairment probably through comparable mechanisms.

Taken together, our results suggest for the first time that the absence of PCSK9 is associated with improved lymphatic function, most likely targeting the collector segment of the vessel. The global knockout of PCSK9 resulted in increased LDLR expression on LEC and in improved lymphatic function of 6-month-old Pcsk9^−/−^ mice compared to WT controls. Collecting lymphatic vessel function is disturbed at a very early stage before atherosclerosis onset and linked to LDLR absence, and this defective transport can be prevented by enhancing VEGF-C/VEGFR-3 specific binding in a cholesterol-independent manner. Although the related specific mechanisms on how LDLR signalling might affect LEC function remain to be understood, our data suggest that LDLR *per se* could have a functional role in the early stage of lymphatic dysfunction, thus corroborating a novel pleiotropic effect of PCSK9 in arterial disease. We believe that our report unveils new important information regarding the specific interactions between the lymphatic system and cardiovascular diseases, thus opening doors to potential therapeutic targets for the prevention and treatment of atherosclerosis.

## Materials and Methods

### Mice

C57BL/6 wild-type (WT) and Pcsk9^−/−^ mice (backcrossed on a C57BL/6 background for 10 generations at our facility) were from Jackson Laboratories. A knockout/transgenic dyslipidemic Ldlr^−/−^; hApoB100^+/+^ mouse colony was established by one of our collaborators at the Montreal Heart Institute (Dr. Éric Thorin)^48^,^49^. All experiments were carried out on 3-, 6- and 12-month-old male and female mice. Differences between sexes were not observed. Animals were housed in a pathogen-free environment under a 12 hours light-dark cycles with free access to water and to standard chow diet. Mice were euthanized with CO_2_ and perfused with 15 ml phosphate buffered-saline (PBS). All experiments were performed in accordance with the Canadian Council on Animal Care guidelines and approved by the Montreal Heart Institute Animal Care Committee.

### Experimental setup

Lymph nodes (LNs), ears, aortas, hearts and popliteal collecting lymphatic vessels were harvested and either freshly processed for flow cytometry analysis and/or western blots, or fixed in 4% paraformaldehyde (PFA) for future analysis, as described below. In some experiments, 3 month-old Ldlr^−/−^; hApoB100^+/+^ mice received an intraperitoneal cavity injection of 25 ng VEGF-C 152s (purified recombinant Rat VEGFC protein (152s), Fitzgerald) dissolved in PBS, three times a week for 4 weeks. The control group received PBS alone.

### Lymphatic Functional Assessment

Lymphatic function was first assessed using Evans blue dye for tracing the path of lymph through popliteal LVs^64^. Mice were anesthetized with isoflurane and following Evans Blue intradermal injection in the footpad, popliteal collecting lymphatic vessels were visualized using a Stereo Discovery V8 (Zeiss). Second, migration of dendritic cells to LN was evaluated after epicutaneous application of FITC solution as described previously^65^. The animals were sacrificed 18 hours later and corresponding skin-draining LNs were recovered and enzymatically digested in collagenase D for 25 min at 37 °C. Cells were then passed through a 70 μm cell strainer, washed, counted, and stained for analysis by flow cytometry (BD Biosciences LSR II). Conjugated antibodies CD11b PerCp-Cy5.5, CD11c PeCy7 and CD45-APC were used (BioLegend). Third, lymphatic function was assessed by quantifying the dermal clearance of dextran by the initial lymphatics, as described previously^66^. Briefly, a total of 1 μl fluorescent (Cy5) dextran (70 kDa) at a concentration of 2 mg/ml in sterile PBS was injected intradermally in the ear pinnae of anesthetized mice. Due to its large size, the tracer is specifically uptaken by blind-ended lymphatic capillaries avoiding absorption by blood capillaries. Fluorescence decay was observed through the skin using a fluorescence stereomicroscope (Leica M205) and images of the skin were acquired every minute for 30 minutes. The rate of clearance was determined by calculating the area under the curve (AUC) of fluorescence intensity at each time point, and normalized to the initial value. The normalized rate of fluorescence decay was then calculated from the slope of AUC *vs.* time, which is considered proportional to the actual rate of dextran-Cy5 clearance.

### Primary mouse lymphatic endothelial cell immunofluorescence and immunoblotting analysis

Popliteal collecting lymphatic vessels were identified following Evans blue dye intradermal injection as described above, and harvested. For analysis of LDLR expression on mouse primary lymphatic endothelial cells, popliteal collecting lymphatic vessels were digested and proteins were extracted following incubation with radioimmunoprecipitation assay (RIPA) buffer containing protease and phosphatase inhibitors. Proteins from lymphatic vessels (30 μg) were separated by 10% sodium dodecyl sulfate-polyacrylamide gel electrophoresis (SDS-PAGE) followed by its transfer to nitrocellulose membranes. The membranes were blocked with 5% nonfat dry milk for 1 hour at room temperature. An antibody against LDLR (Novus Biologicals) was incubated with the membranes overnight at 4 °C, and HRP-conjugated secondary antibodies were used for detection using the Western Lightning Ultra chemiluminescence kit (PerkinElmer). Experiments were performed by pooling of 3–4 mice *per* experimental group. The presence of LDLR was also confirmed on isolated popliteal lymphatics of mice by whole-mount immunofluorescence analysis following incubation with anti-podoplanin (Angio Bio Co.) and anti-LDLR antibodies. Images were acquired with an LSM 710 Confocal Microscope (Zeiss) equipped with a 63x/1.4 oil dic objective. Images were deconvolved theoretical point spread function using Huygens Professional software (Scientific Volume Imaging) and volumes were rendered using the surface renderer option.

### Quantification of initial lymphatic density

The heart and aorta were removed and fixed in 4% PFA for 2 hours.The heart was transferred into PBS containing 30% sucrose (wt/vol) overnight at 4 °C before being immersed in OCT compound and stored at −80 °C. Eight-micrometer-thick cryosections of the aortic sinus were prepared. Cross-sections of the aortic sinus were stained with anti-LYVE-1 (ABCAM) and anti-CD68 (Biolegend) antibodies, and then incubated with the appropriate secondary antibodies. As macrophages can also be positive for LYVE-1, adventitial lymphatic capillaries were identified as LYVE-1^+^ CD68^−^ cells forming vessel-like shapes. Whole-mount immunohistochemical analysis of the ear dermis to visualize lymphatic vessels was performed as described previously^47^. Ear dermis were stained for lymphatic capillaries (anti-LYVE-1, ABCAM) at 4 °C, and then sections were incubated with Alexa Fluor 647 conjugated donkey anti-rabbit antibody and Cy3 donkey anti-rat (Jackson ImmunoResearch). All imaging was performed on a Fluoview FV10i (Olympus). All vessel counts were performed by one observer. The relative quantification of the number of lymphatic capillaries (LYVE-1^+^ vessels), their diameter and the total surface area they occupy was determined by computer-assisted morphometric analysis. Neutral lipid assessment in atherosclerotic lesions was performed by Oil-red-O (ORO) staining (Sigma).

### Immunohistochemistry

The back skin of the animals was shaved and harvested, fixed in 10% formalin, and embedded in paraffin. Eight-micrometer thick back-skin sections were stained with Hematoxylin and eosin (H&E). Pictures were taken with an Olympus B45 microscope and visualized using ImagePro Plus 7.0 software. All image handling was performed using ImageJ software.

### Thoracic Duct Cannulation

Mice were anesthetized with isoflurane (4% for induction, 2–3% for maintenance). The anesthetized animal was positioned on its right side and a cannula was inserted into the thoracic lymph duct above the cisterna chyli between the transverse lumbar artery and the diaphragm. Lymph was collected continuously for 30–45 minutes with a tube attached to a syringe coated with EDTA 0.1 M. Collected lymph was centrifuged at 1200 g for 10 minutes. To avoid thawing-related damage on lipoprotein conformation, sucrose (5%) was added before samples were stored at −80 °C for further batch analysis.

### Measurement of total cholesterol, HDL and PCSK9

Blood was collected on heparin by cardiac puncture and plasma was obtained following centrifugation at 2400 g for 10 minutes and sucrose was added before samples were stored at −80 °C. Batch analysis was performed to measure circulating total cholesterol (Wako), HDL (Siemens Healthcare Diagnostics, HDL Flex reagent cartridge) and PCSK9 (ELISA, Circulex) in plasma and lymph according to the manufacturer’s protocols.

### Statistics

Data are expressed as the mean ± SEM. Statistical differences were assessed using a two-tailed parametric or non-parametric Student’s *t* test, unless otherwise stated, with p < 0.05 reported as statistically significant, using Prism software version 6.0 c (GraphPad).

## Additional Information

**How to cite this article**: Milasan, A. *et al*. Effects of LDL Receptor Modulation on Lymphatic Function. *Sci. Rep.* **6**, 27862; doi: 10.1038/srep27862 (2016).

## Supplementary Material

Supplementary Information
