# Supplementary material for: Effects of LDL Receptor Modulation on Lymphatic Function
Source: Sci Rep. 2016 Jun 9;6:27862. doi: 10.1038/srep27862 (PMC4899717; doi:10.1038/srep27862)
Supplement: Supplementary Information [file srep27862-s1.pdf]

Effects of LDL Receptor Modulation on Lymphatic Function

Andreea Milasan, François Dallaire, Gaétan Mayer and Catherine Martel

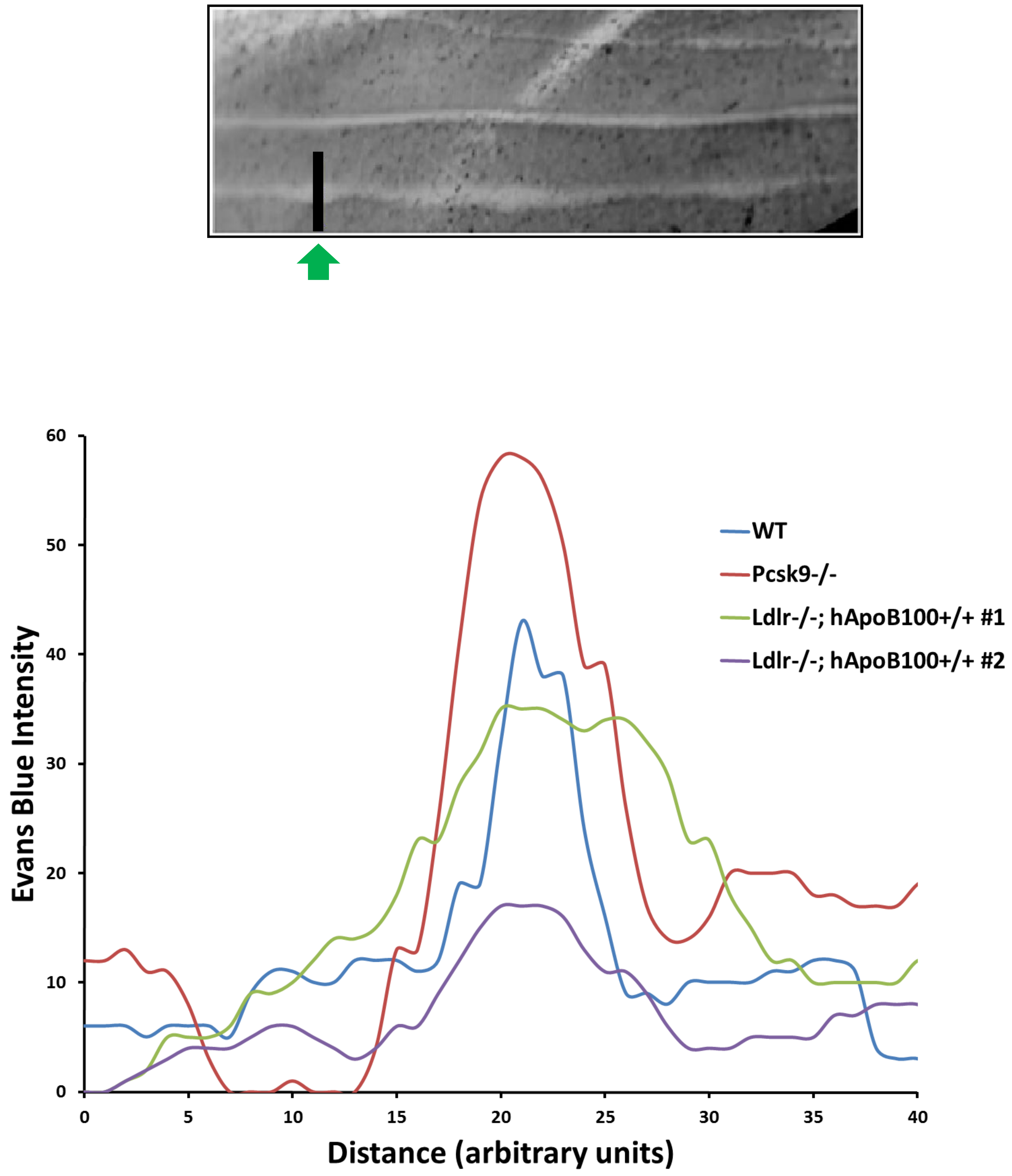

Supplementary figure 1. *In vivo* semi-quantitative visualization of Evans Blue dye transported through a popliteal lymphatic collecting vessel. Lymphatic function was assessed following Evans Blue dye intradermal injection in mice footpads. After 30 minutes, the dye intensity distribution perpendicular to the popliteal vessels was assessed using ImageJ (as illustrated by the green arrow, upper panel). The histogram (lower panel) illustrates the different intensities of the WT (blue), Pcsk9$^{-/-}$ (red) and the two different Ldlr$^{-/-}$; hApoB100$^{+/+}$ mice collecting vessel phenotypes observed in figure 1A (i.e. leaky (green) and dye-free (purple) collecting vessel segments. Experiments were performed using 3 replicates per experimental group.

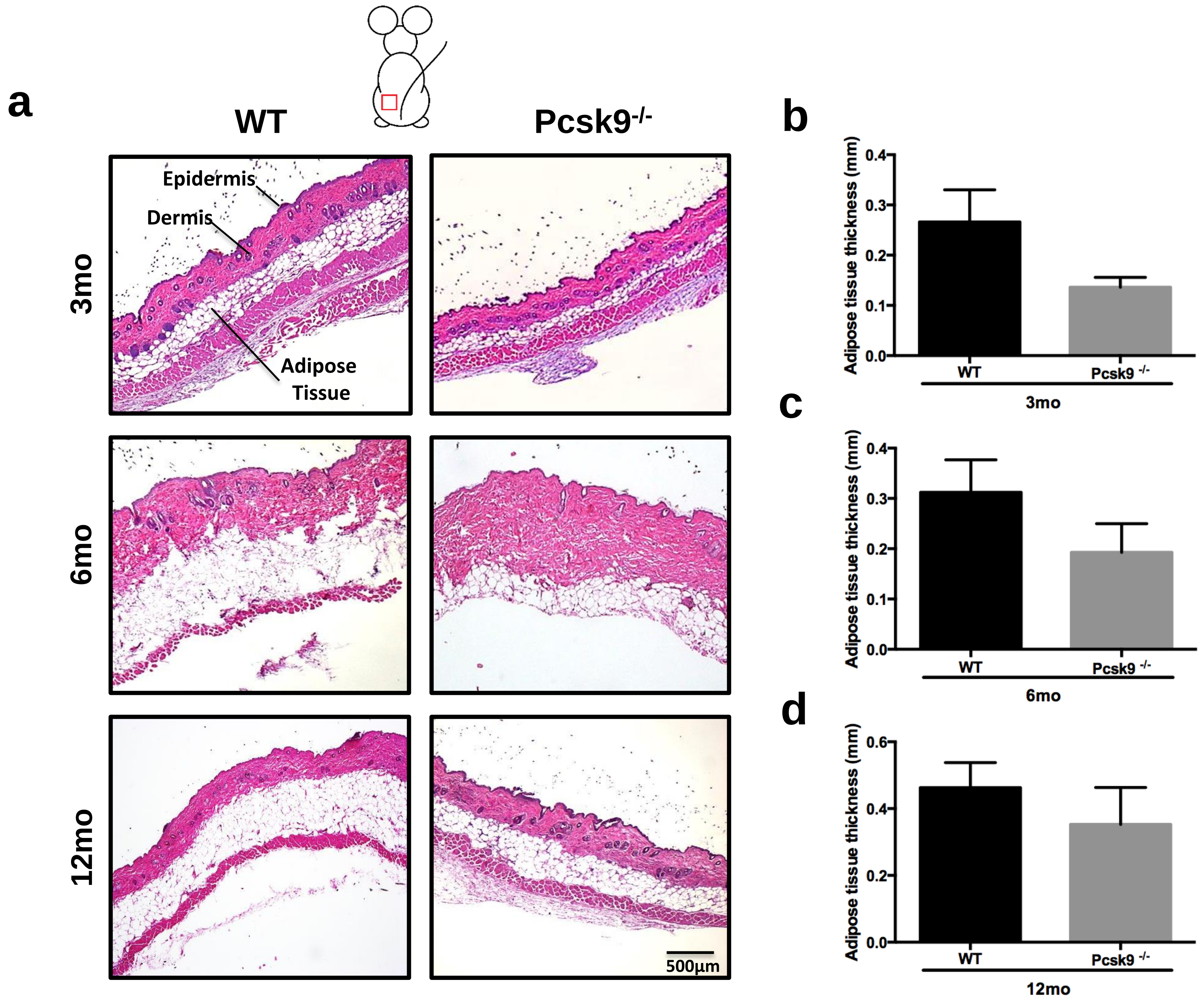

Supplementary figure 2. Adipose tissue layer from back skin in Pcsk9$^{-/-}$ and WT mice. (a) Hematoxylin and eosin staining was performed on 8 µm-thick paraffin skin sections of 3-, 6- and 12-month-old WT and Pcsk9$^{-/-}$. Quantification of adipose tissue thickness in back skin is illustrated at **(b)** 3-, **(c)** 6- and **(d)** 12-month-old mice. Experiments were performed with 3-4 replicates per experimental group (mean ± SEM). Picture were taken with an Olympus B45 microscope and analyzed using ImagePro Plus 7.0 software. All image handling was performed using ImageJ software.

a b

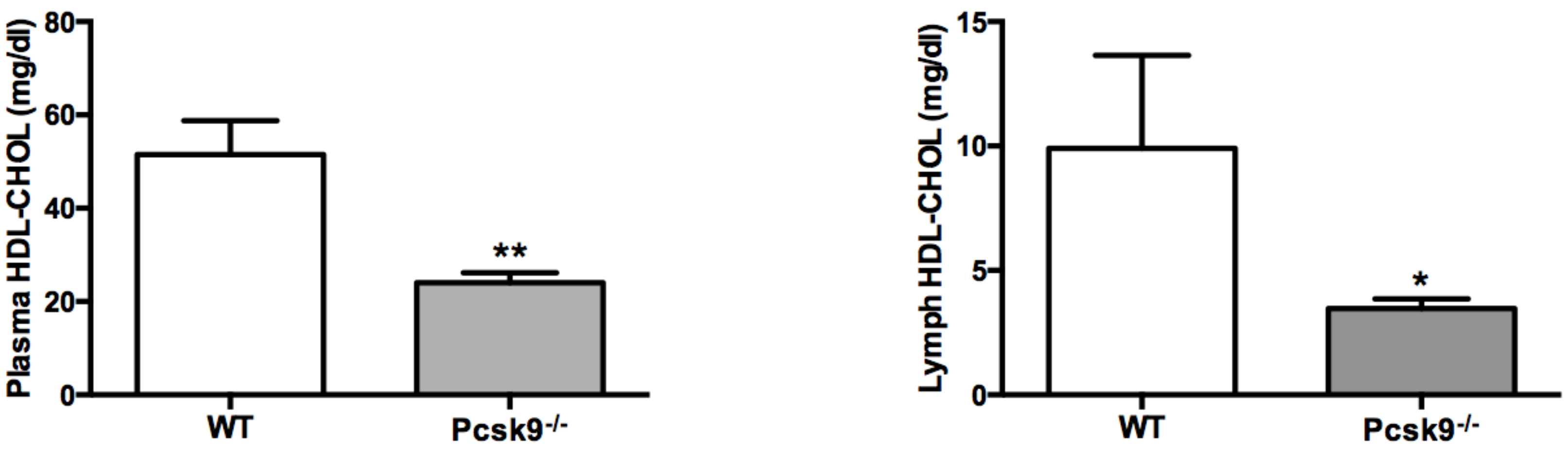

Supplementary figure 3. Cholesterol profile in our different mouse models. Both **(a)** plasma and **(b)** lymph HDL-C were analyzed in 6-month-old WT and Pcsk9$^{-/-}$ mice. Experiments were performed with 4-7 mice per experimental group. *$p<0.05$ and **$p<0.01$.

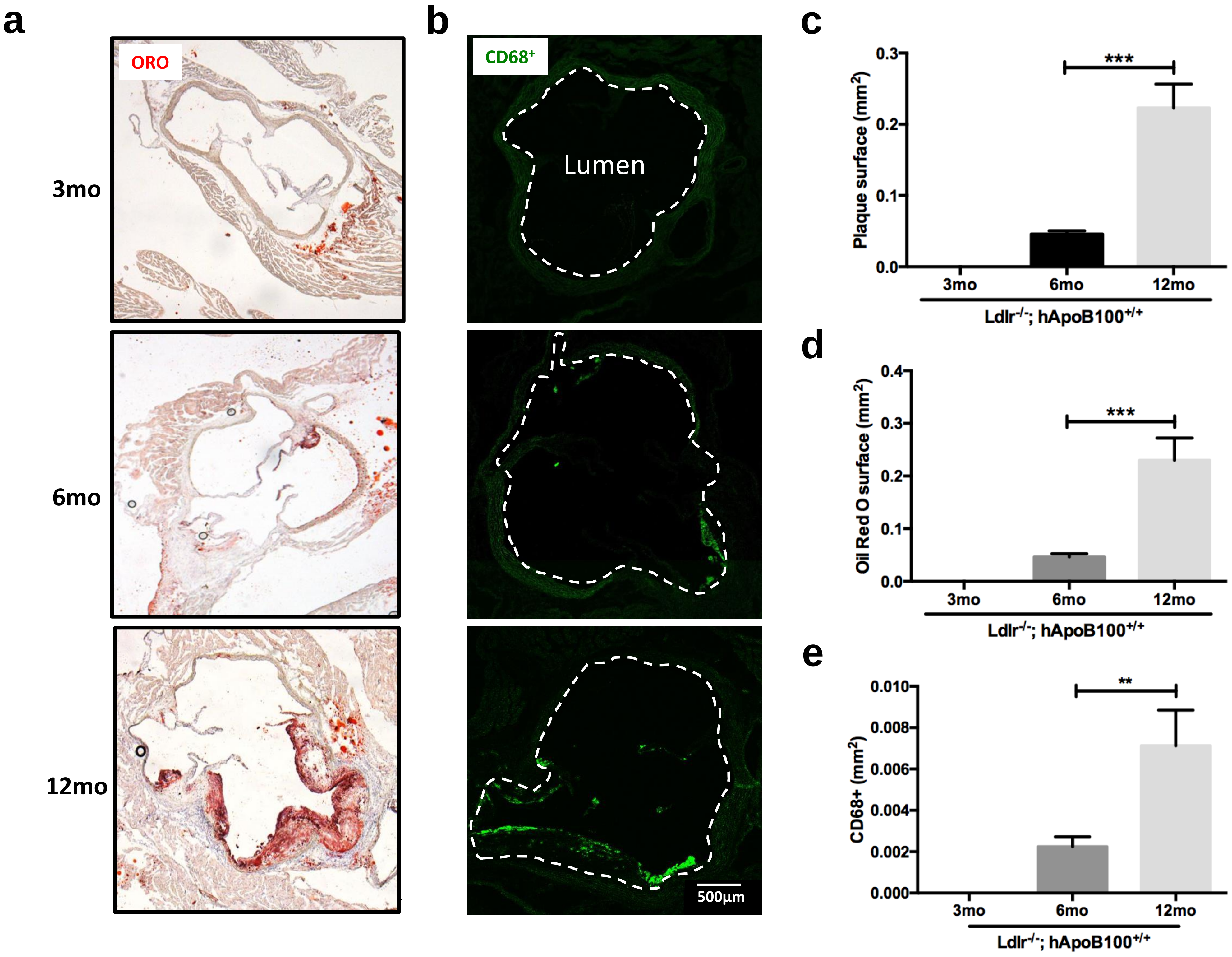

Supplementary figure 4. Plaque development during atherosclerosis progression in Ldlr$^{-/-}$; hApoB100$^{+/+}$ mice. Hearts were harvested and immersed in OCT compound. **(a)** Neutral lipid- (Oil Red O) and **(b)** macrophage (CD68^{+})-positive areas were quantified in 8 µm-think aortic sinus sections of 3-, 6- and 12-month-old Ldlr$^{-/-}$; hApoB100$^{+/+}$ mice. **(c)** Plaque surface (mm^{2}), **(d)** Oil Red O and **(e)** CD68^{+} area were quantified using ImageJ software. Experiments were performed with 4-9 mice per experimental group. $^{**}p<0.01$ and $^{***}p<0.001$.

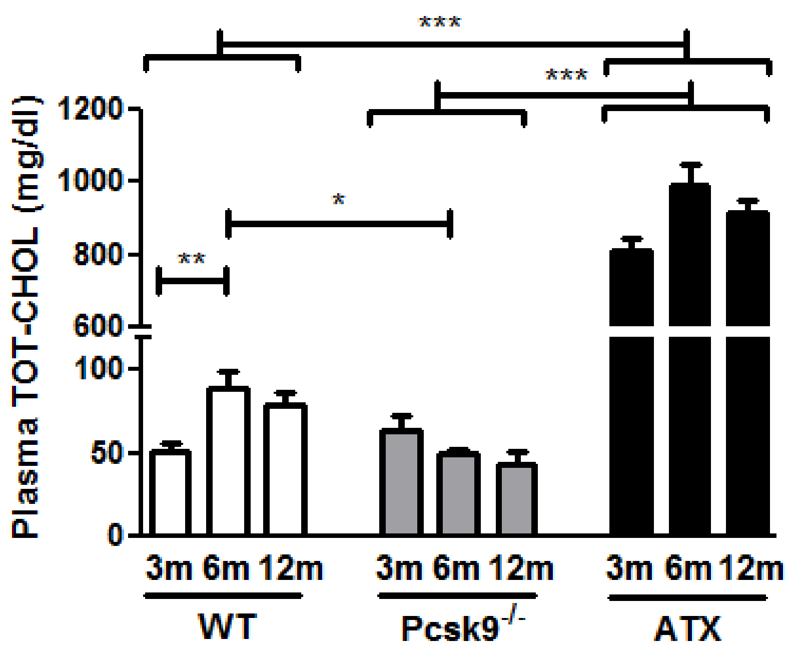

Supplementary figure 5. Cholesterol profile in our different mouse models. Plasma total cholesterol at 3-, 6- and 12-months old WT, Pcsk9$^{-/-}$ and Ldlr$^{-/-}$; hApoB100$^{+/+}$ mice. Experiments were performed with 4-7 mice per experimental group. *$p<0.05$, **$p<0.01$ and ***$p<0.001$.
